# Analysis of putative resistance gene loci in UK field populations of *Haemonchus contortus* after 6 years of macrocyclic lactone use

**DOI:** 10.1016/j.ijpara.2016.03.010

**Published:** 2016-09

**Authors:** Roz Laing, Kirsty Maitland, Lenka Lecová, Philip J. Skuce, Andy Tait, Eileen Devaney

**Affiliations:** a Institute of Biodiversity, Animal Health and Comparative Medicine, College of Medical, Veterinary and Life Sciences, University of Glasgow, Scotland, UK; b Department of Tropical Medicine, First Faculty of Medicine, Charles University in Prague, Czech Republic; c Moredun Research Institute, Pentlands Science Park, Penicuik, UK

**Keywords:** Anthelmintic resistance, Parasite, Nematode, Sheep, Macrocyclic lactone, Ivermectin

## Abstract

Sheep farmers in the UK rely on strategic anthelmintic use to treat and control gastrointestinal roundworms in their flocks. However, resistance to these drugs is now widespread and threatens the sustainability of sheep production. The mechanisms underlying resistance to the most commonly used class, the macrocyclic lactones, are not known and sensitive diagnostic tools based on molecular markers are not currently available. This prohibits accurate surveillance of resistance or assessment of strategies aimed at controlling its spread. In this study, we examined four UK field populations of *Haemonchus contortus*, differing in macrocyclic lactone treatment history, for evidence of selection at ‘candidate gene’ loci identified as determining macrocyclic lactone resistance in previously published research. Individual worms were genotyped at *Hc-lgc-37*, *Hc-glc-5, Hc-avr-14* and *Hc-dyf-7*, and four microsatellite loci. High levels of polymorphism were identified at the first three candidate gene loci with remarkably little polymorphism at *Hc-dyf-7.* While some between-population comparisons of individual farms with and without long-term macrocyclic lactone use identified statistically significant differences in allele frequency and/or fixation index at the *Hc-lgc-37*, *Hc-glc-5* or *Hc-avr-14* loci, we found no consistent evidence of selection in other equivalent comparisons. While it is possible that different mechanisms are important in different populations or that resistance may be conferred by small changes at multiple loci, our findings suggest that these are unlikely to be major loci conferring macrocyclic lactone resistance on UK farms or suitable for diagnostic marker development. More powerful approaches, using genome-wide or whole genome sequencing, may be required to define macrocyclic lactone resistance loci in such genetically variable populations.

## Highlights

• Field populations of *Haemonchus contortus* were collected from UK sheep farms.
• Farms with 6 years of macrocyclic lactone (ML) use or avoidance were compared.
• Parasites were genotyped at microsatellite loci and putative ML resistance loci.
• No evidence of ML selection was detected at *Hc-lgc-37*, *Hc-glc-5, Hc-avr-14* or *Hc-dyf-7.*
• These loci are unlikely to be suitable for diagnostic marker development in the UK.

## Graphical abstract

**Figure:**
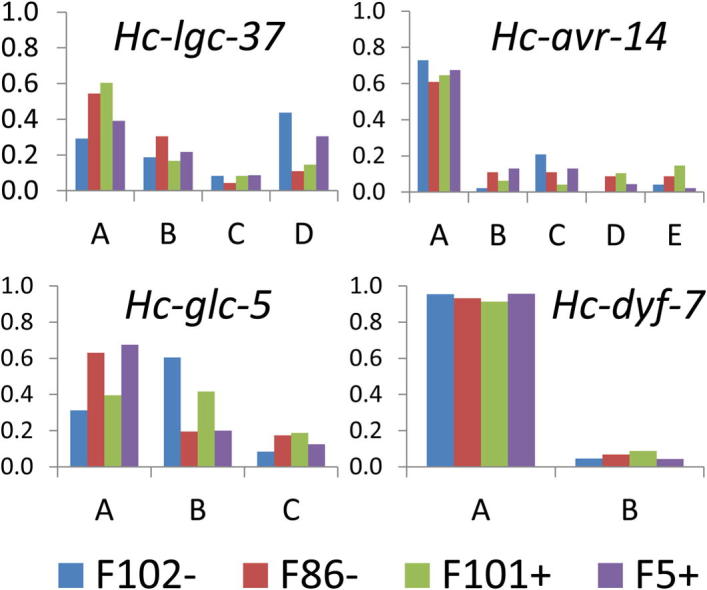

## 1 Introduction

The parasitic nematodes that infect grazing livestock are ubiquitous and impact heavily on animal welfare and production. Treatment of clinical cases depends on the use of anthelmintics and most control programmes rely on strategic dosing with the same drugs. However, anthelmintic resistance is now widespread and is considered to be a significant threat to global food security (Kaplan and Vidyashankar, 2012). Currently, the impact of resistance is most apparent in the sheep sector, where it is a major constraint on production and threatens the industry worldwide.

In the UK, 93–99% of sheep farmers routinely use anthelmintics (Burgess et al., 2012, Morgan et al., 2012). Five classes of anthelmintic are licensed for use in sheep, of which the most widely used is the macrocyclic lactone (ML) group (Burgess et al., 2012, Morgan et al., 2012), most commonly ivermectin (IVM) or moxidectin (MOX). Accurate assessment of the prevalence of resistance to the MLs is limited by the lack of a sensitive test, but there are numerous reports of ML-resistant parasites in Scottish sheep flocks (Sargison et al., 2005, Sargison et al., 2007, Sargison et al., 2010) and two small-scale studies have detected ML resistance on 35–63% of UK sheep farms tested (Bartley et al., 2006, Stubbings and Scops, 2012). Worldwide, ML resistance is now widespread in parasites of sheep, goats, cattle and horses (reviewed in Kaplan and Vidyashankar (2012)). In humans, IVM is used to treat infection by the filarial parasite *Onchocerca volvulus* (the cause of Onchocerciasis or River Blindness) and reports of treatment failures have raised concerns that resistance may be emerging in this species too (Osei-Atweneboana et al., 2007, Osei-Atweneboana et al., 2011).

In parasitic nematodes, the mechanisms underlying resistance to the MLs are not fully understood and sensitive diagnostic tools, based on molecular markers, are not currently available. This prohibits accurate surveillance of resistance or assessment of the impact of different control strategies. In the free-living nematode *Caenorhabditis elegans*, the biological targets of the MLs are the glutamate-gated chloride channels (Cully et al., 1994, Hibbs and Gouaux, 2011) but the mechanism of resistance appears to be complex. In 2000, Dent et al. reported that simultaneous mutation of at least two of three genes encoding glutamate-gated chloride channel subunits (*glc-1*, *avr-14* and *avr-15*) was required to confer significant resistance to IVM. The level of resistance was further modulated by a number of genes acting in the nematode nervous system including innexins (*unc-7* and *unc-9*) and a dye filling defective (dyf) gene *osm-1*. Subsequently, Ghosh et al. (2012) identified a four amino acid deletion in the ligand-binding domain of GLC-1 conferring abamectin resistance in multiple diverse natural populations of *C. elegans*. However, a number of resistant populations were found to lack the deletion. Further, when six abamectin-resistant populations that did encode the deletion were crossed with N2 worms containing wild type *glc-1*, the F1 progeny of four crosses regained ML sensitivity, but two remained resistant, suggesting the presence of a second dominant resistance locus. More recently, Urdaneta-Marquez et al. (2014) described a *dyf-7* allele, encoding a truncated protein, which was responsible for IVM resistance in a laboratory-selected isolate of *C. elegans.* Thus, in *C. elegans*, ML resistance can be conferred by different genes and often involves multiple genes, and it is possible that a similar diversity of mechanisms may exist in parasitic nematodes.

We have taken a population genetics approach to define whether a series of candidate ML resistance genes are under selection in *Haemonchus contortus*, an economically important gastrointestinal parasite of sheep*.* In this species, polymorphisms in a number of genes have been associated with resistance in various laboratory and field isolates (reviewed in Kotze et al. (2014)), but the relevance of these candidate genes to resistance in UK sheep flocks remains unclear. *Haemonchus contortus* lacks an orthologue of *C. elegans glc-1* (Laing et al., 2013), but the parasite-specific *Hc-glc-5* (also known as *HcGluCla*) encodes an IVM, MOX and glutamate-gated chloride channel subunit (Forrester et al., 1999, Forrester et al., 2002). One *Hc-glc-5* allele, detected by single strand conformation polymorphism (SSCP) analysis, was found at a higher frequency in IVM and MOX selected laboratory isolates relative to unselected isolates derived from the same parental populations (Blackhall et al., 1998a); more recent studies have found no significant coding differences in *Hc-glc-5* in two IVM-resistant isolates compared with sensitive isolates (Cheeseman et al., 2001, Williamson et al., 2011). *Hc-avr-14* (also known as *gbr-2* or *HcGluClα3*) is the *H. contortus* orthologue of *C. elegans avr-14.* In both species, the gene is alternatively spliced to encode two glutamate-gated chloride channel subunits, *avr-14a* and *avr-14b,* with the *avr-14b* subunit binding IVM (Dent et al., 1997, Jagannathan et al., 1999, Cheeseman et al., 2001). In *Cooperia oncophora*, an *avr-14b* allele encoding a L256F substitution confers two-to-three fold resistance (Njue et al., 2004). While this allele was present at a higher frequency in one IVM-resistant isolate compared with one sensitive isolate of *C. oncophora* (Njue and Prichard, 2004), it was not found in three other IVM-resistant isolates, although lower allelic diversity was observed at this locus relative to a susceptible isolate (El-Abdellati et al., 2011). In *H. contortus*, a T300S substitution in AVR-14B has been reported in the ML-resistant White River Strain (WRS), but no channels were formed by the expression of *Hc-avr-14b* T300S in *Xenopus* oocytes and the mutation had no effect on IVM binding (McCavera et al., 2009). However, the same study found channels were formed by the expression of *Hc-avr-14b* L256F (the substitution identified in *C. oncophora*) and the mutation reduced IVM binding relative to wild type. The *H. contortus* orthologue of *C. elegans lgc-37* (also known as *hg1*) encodes a γ-aminobutyric acid (GABA)-gated chloride channel subunit (Laughton et al., 1994, Feng et al., 2002). One *Hc-lgc-37* allele, detected by SSCP analysis, was found at a higher frequency in an IVM selected laboratory isolate than in the unselected isolate derived from the same parental population, while a different *Hc-lgc-37* allele was found at a higher frequency in an IVM and MOX selected laboratory isolate (Blackhall et al., 2003). The putative resistance allele in the IVM and MOX selected isolate encodes a K169R and a Q176L substitution in the cys-loop region and a V436I and a H442Y substitution in the membrane-spanning region (Feng et al., 2002). Recently, selection at the *Hc-dyf-7* locus has been reported in IVM and MOX selected laboratory isolates of *H. contortus* (Urdaneta-Marquez et al., 2014). Fifteen SNPs in the coding sequence of *Hc-dyf-7* are proposed to correlate with ML resistance in *H. contortus* isolates from various countries, but three of these polymorphisms (A141G, T234C and G438T) associate with all resistant isolates and are found to most reliably predict resistance status. Resistance-associated mutations have also been described in a dopamine-gated chloride channel subunit (Rao et al., 2009) and previous studies reported selection at a P-glycoprotein locus in IVM and MOX selected strains of *H. contortus* (Blackhall et al., 1998b). Increased P-glycoprotein expression has also been described in various anthelmintic-resistant isolates of *H. contortus* (Prichard and Roulet, 2007, Williamson et al., 2011). Thus, alleles of several genes whose products have a potential functional role in ML action or efflux have been identified as associated with the resistant phenotype - the so called ‘candidate’ gene loci.

The concept underlying these studies is that a beneficial mutation should increase in frequency under selection, so the prevalence of an allele associated with resistance should be higher in the resistant population than the susceptible population. A limitation of this approach is the reliance on comparisons between populations of worms that may differ not only in their resistance status, but may, for example, exhibit geographical differences or have undergone bottlenecks on selection. Such genetic differentiation could generate significant differences at loci unrelated to resistance, which would only be detected with the use of neutral markers, but these have not frequently been used. Further, the high levels of genetic diversity in populations of *H. contortus* may be very likely to produce stochastic differences in standing genetic variation at almost any locus examined in isolation, again emphasising the need for analysis of multiple, preferably neutral, markers. The majority of studies to date have also used laboratory isolates of *H. contortus* or strains selected for ML resistance in the laboratory, so the results may not be representative of the situation in the field.

In this study, we examined four UK field populations of *H. contortus*, differing in ML treatment history, for evidence of selection at four candidate gene loci (*Hc-glc-5, Hc-avr-14*, *Hc-lgc-37* and *Hc-dyf-7*). In light of the high levels of polymorphism reported in nematode populations, we chose to look for evidence of selection at each genomic locus rather than focusing on any specific mutation. *Haemonchus contortus* populations on three of the four farms have been previously characterised with 10 microsatellite markers (Redman et al., 2015) and four of these markers were included in this study. Our objectives were to assess the level of polymorphism in four candidate genes in field populations of *H. contortus*, and to test for evidence of selection for specific alleles or combinations of alleles after 6 years of ML treatment.

## 2 Materials and methods

### 2.1 Parasite populations from UK farms

In 2008, farmers from 118 sheep farms throughout the UK completed a questionnaire detailing anthelmintic use for the current year and previous 5 years, and submitted a pooled faecal sample from 20 ewes (Burgess et al., 2012). Eggs were extracted from these samples and incubated to the L1 stage prior to storage in 70% ethanol at −80 °C. Parasite populations from this ‘biobank’ were selected based on prevalence of *H. contortus* and farm history of ML use. Four *H. contortus*-positive farms were chosen for analysis (Table 1): two ML+ farms with frequent use of MLs in ewes and lambs for 6 years (F101+ and F5+), one ML− farm with no use of MLs in ewes or lambs for 6 years (F102−), and one ML− farm with no use of MLs in ewes or lambs except at quarantine, which as a closed flock would be a rare occurrence (F86−). The ML+ farms practiced behaviour that would be deemed risky for ML resistance: both used a long-acting ML in ewes at lambing, and dosed based on an estimate of weight. The ML− farms did not use a long-acting ML in ewes at lambing and dosed to the heaviest animal. One farm from each group practiced dose and move, and one from each group did not.

### 2.2 DNA lysate preparation and identification of *H. contortus* larvae

DNA lysates were made from individual L1s from year six (2008) ewes from each of the four farms. Briefly, individual L1s were plated into 96 well plates containing 20 μl of lysis reagent (1000 μl of cell lysis buffer (Viagen, USA), 50 μl of DTT and 50 μl of proteinase K (Qiagen, UK) per well and incubated at 60 °C for 2 h, then held at 85 °C for 45 min to denature the proteinase K. The DNA lysates were diluted (1 in 20) in nuclease-free water and aliquots were stored at −80 °C. *Haemonchus contortus* larvae were identified from each farm using species-specific PCR, based on amplification of the *H.* contortus internal transcribed spacer 2 (ITS-2) region (Redman et al., 2008), and used for subsequent genotyping.

### 2.3 Candidate ML resistance loci sequencing and genotyping

Candidate ML resistance genes in *H. contortus* were identified from the literature. Coding sequences for *Hc-glc-5*, *Hc-avr-14* and *Hc-dyf-7* GenBank Accession numbers AF034609, Y14234, KF927016) were aligned to the *H. contortus* genome sequence (Laing et al., 2013) and primers were designed to amplify their genomic loci. Published primers were available for *Hc-lgc-37* (Blackhall et al., 2003). Supplementary Table S1 shows all primer sequences and amplicon sizes.

Candidate gene loci were PCR-amplified (Phusion polymerase, New England Biolabs, UK) from a pooled sample of genomic DNA from 20 L1s from farm F102−. The PCR products were cloned (Zero Blunt TOPO PCR Cloning Kit, ThermoFisher, UK) and sequenced in both directions (Eurofins MWG, Germany). The sequences were aligned in Geneious version 6.1.2 (http://www.geneious.com) and used to design discriminatory restriction fragment length polymorphism (RFLP) digests and allele-specific PCRs for individual worm genotyping.

Twenty-three or 24 individual L1s from farms F102−, F86−, F101+ and F5+ were genotyped at the four candidate gene loci. *Taq*I (New England Biolabs) enzymatic digests were used to genotype individuals at the *Hc-lgc-37*, *Hc-glc-5* and *Hc-dyf-7* loci, using semi-nested PCR products for *Hc-glc-1* and *Hc-dyf-7* (Supplementary Table S1). Supplementary Fig. S1 demonstrates the allele scoring approach, using the *Hc-glc-5* locus as an example. The *Taq*I cut sites are highlighted in the F102- sequences (Supplementary Fig. S1A) and a typical gel of RFLP digests is shown in Supplementary Fig. S1B. Alleles with unclear or novel profiles were resolved by sequencing, as were a random subset for quality control.

An allele-specific PCR for the most common *Hc-avr-14* allele A (Supplementary Table S1; primers Fs and RsA) and for non-A alleles (Supplementary Table S1; primers Fs and RsnA), followed by sequencing of the latter, was used to resolve genotypes at the *Hc-avr-14* locus (Supplementary Fig. S2). Five clones were sequenced to confirm a non-A homozygote. For all candidate gene loci, rare alleles (frequency <0.08) were grouped for population genetic analysis.

To further characterise the *Hc-dyf-7* locus, pooled lysates from 20 L3s were prepared for three laboratory isolates: one ML-susceptible isolate, MHco3(ISE) (Roos et al., 2004), and two ML-resistant isolates, MHco4(WRS) (van Wyk and Malan, 1988) and MHco10(CAVR) (Le Jambre et al., 1995). The *Hc-dyf-7* locus was PCR amplified and cloned (as described above) from the three isolates, and five clones were sequenced for each.

### 2.4 Microsatellite marker analysis

Previously published microsatellite markers 22co3, Hcms25, Hcms36 and 8a20 were amplified for each individual L1 as described by Redman et al. (2008). Microsatellites were sized on an ABI Prism 3130xl Genetic Analyser (ThermoFisher) with a GeneScan ROX 400HD size standard (ThermoFisher) and analysed with GeneMapper (ThermoFisher).

### 2.5 Population genetic analysis

Arlequin version 3.5.1.3 (Excoffier et al., 2005) was used to measure heterozygosity, allele richness, Hardy–Weinberg Equilibrium (HWE) and linkage disequilibrium (LD). Analysis of Molecular Variance (AMOVA) was used to assess the partitioning of genetic variation within and between populations. Null corrections for fixation index (FST) estimates were calculated with FreeNA (Chapuis and Estoup, 2007). Allele frequency graphs and Principal Co-ordinates Analysis (PCoA) plots were generated in Excel with a Genalex plug-in (Peakall and Smouse, 2012). *X*^2^ analyses were performed to test for allele frequency differences. LOSITAN (Antao et al., 2008) was used for FST outlier analysis; 50,000 simulations were run to estimate the mean FST then again to identify FST outliers, using an infinite alleles model.

## 3 Results

### 3.1 Genetic diversity at candidate gene loci

Twenty *H. contortus* L1s from F102− were sequenced at the candidate gene loci to assess standing genetic variation in an unselected population and to design an efficient genotyping method for each locus. This revealed high levels of genetic diversity, both in terms of allele richness and sequence variation, at three candidate gene loci (*Hc-lgc-37*, *Hc-glc-5* and *Hc-avr-14*) (Supplementary Fig. S3). Numerous single nucleotide polymorphisms (SNPs) and indels were observed at all loci (minimum identity of bases in all pairwise allele comparisons were 80.4% at *Hc-glc-5*, 89.3% at *Hc-avr-14,* and 90.3% at *Hc-lgc-37*) but all mutations in coding regions were synonymous. There was notably little genetic diversity at the *Hc-dyf-7* locus, which is described below. The genomic sequences were used to design RFLP screens for three loci (*Hc-lgc-37*, *Hc-glc-5* and *Hc-dyf-7*) and an allele-specific PCR for the most common allele at the *Hc-avr-14* locus. Initially various double digests were trialled in an attempt to capture the full spectrum of allele diversity at the *Hc-lgc-37* and *Hc-glc-5* loci, but these proved difficult to interpret and, as rare alleles would be grouped for analysis, a simpler single digest with *Taq*I was chosen as an alternative.

### 3.2 Genetic differentiation between farm populations

**Fig. 1:**
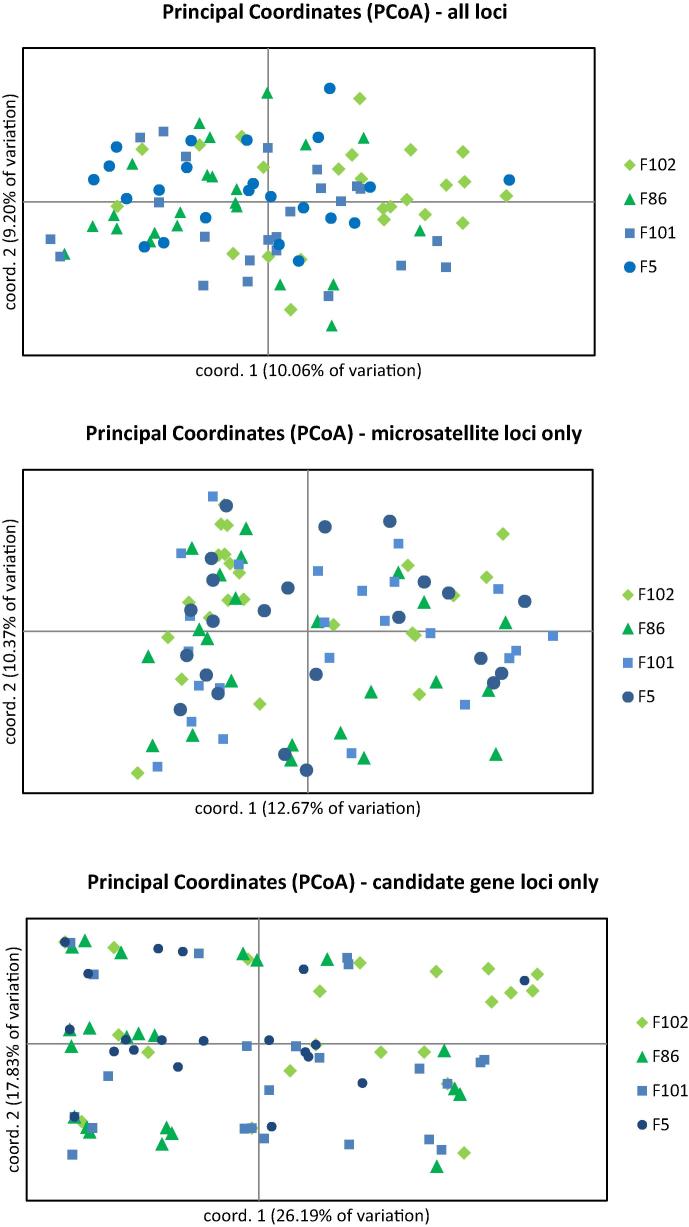
Principal Co-ordinate Analysis plots. The plots show individual *Haemonchus contortus* L1s from field populations without a history of macrocyclic lactone use (F102− and F86−) and field populations with a history of frequent macrocyclic lactone use (F101+ and F5+). The plots show little differentiation between individuals from different farm populations, other than some clustering of individuals from F102−, which is consistent with the Analysis of Molecular Variance partitioning of genetic variation and the population fixation index values.

Ninety-four individual *H. contortus* L1s from four farms (47 ML− and 47 ML+ L1s) were genotyped at the four candidate gene loci and four microsatellite loci. Three of the four farm populations had previously been characterised with 10 microsatellite markers, showing low levels of differentiation between farms (Table 2 and Redman et al. (2015)). F5+ had not been previously characterised. Based on the four microsatellite loci and four candidate genes, population pairwise FSTs showed limited genetic differentiation between F86−, F101+ and F5+ (0.0088–0.0221), with F102− showing the most differentiation from all other farms, but again with modest FST values (0.0362–0.0670). Similarly, the majority of diversity partitioned within populations (97.43%), with only 2.57% partitioning between populations, suggesting these were appropriate populations for between-farm analysis. Fig. 1 shows three PCoA plots of the 94 individual larvae, highlighting the lack of genetic differentiation between farm populations. This finding was further tested by assigning the 94 individuals to four populations probabilistically, based on genotype alone, using the software STRUCTURE (Pritchard et al., 2000). Each of the four software-generated populations contained individuals from all farms, with no single farm contributing a predominant number of individuals to any one group (data not shown), again supporting limited inter-farm differentiation.

### 3.3 Hardy–Weinberg equilibrium

In all populations, genotypes at the *Hc-lgc-37*, *Hc-avr-14* and *Hc-dyf-7* loci were in HWE. *Hc-dyf-7* resides on the X chromosome, so this is likely to represent a type II error, because the locus is almost monomorphic i.e. the lack of observed heterozygotes is not statistically different from the low number predicted. In F86− and F101+ only, *Hc-glc-5* showed significant departure from HWE at *P* < 0.05, with fewer observed heterozygotes than expected. This does not correlate with ML selection. Consistent with previous studies, all four microsatellites showed significant departure from HWE, which is thought to reflect the presence of null alleles inflating the number of observed homozygotes (Chapuis and Estoup, 2007, Redman et al., 2015).

### 3.4 Allele frequency analysis

**Fig. 2:**
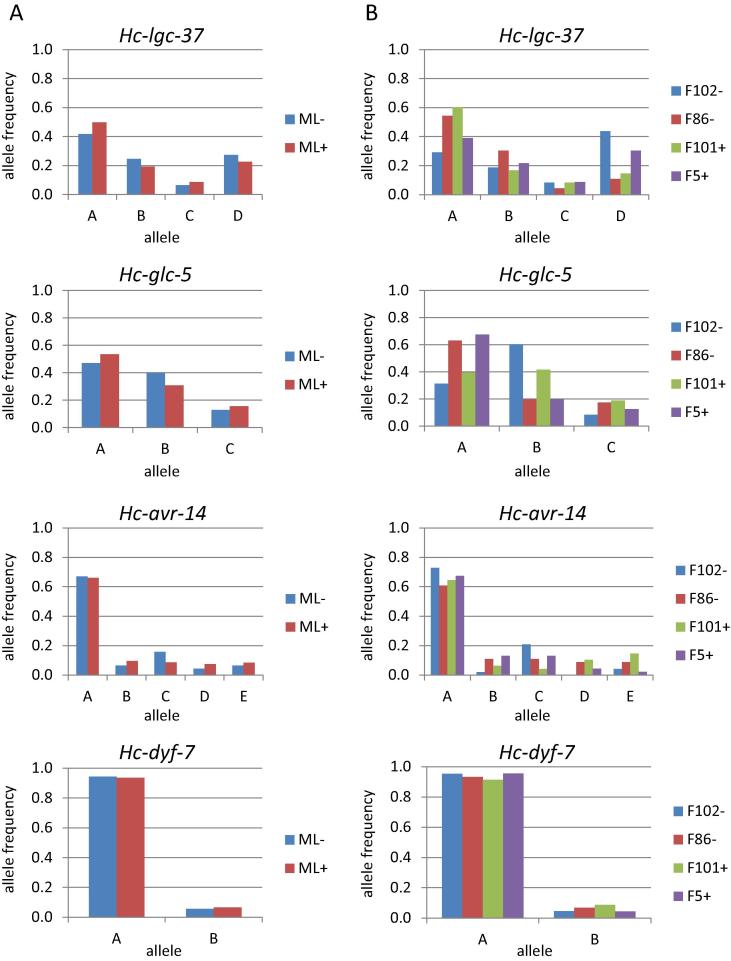
Allele frequencies at four candidate gene loci in *Haemonchus contortus*. Column A shows allele frequencies in farm populations pooled by macrocyclic lactone treatment history. ML−, field populations without history of macrocyclic lactone use; ML+, field populations with a history of frequent macrocyclic lactone use. No significant differences in allele frequencies between ML− and ML+ populations were present. Column B shows allele frequencies in individual farm populations. Significant differences in allele frequencies were observed in some between-farm comparisons but not others. No consistent differences were observed between all ML− farms and all ML+ farms. See Table 3 for *X*^2^ analyses.

Pairwise comparisons of allele frequency identified statistically significant (*P* < 0.05) differences at the *Hc-lgc-37*, *Hc-glc-5* and *Hc-avr-14* loci in a number of between-farm comparisons (Fig. 2, Table 3). While allele frequency differences were apparent in some individual farms with ML selection relative to those without, there were also statistically significant differences between individual farms with no ML selection. For example, allele frequencies at the *Hc-lgc-37* locus differ significantly between F102− and F101+, with a dramatically higher frequency of allele A on F101+, which could be interpreted as indicative of ML selection. However, this finding was not observed in any other ML− and ML+ farm comparison and a similar, statistically significant, difference in *Hc-lgc-37* allele frequency was observed in a comparison of F102− with another unselected farm, F86−. Similarly, significant differences in allele frequencies at the *Hc-glc-5* and *Hc-avr-14* loci were observed in various pairwise comparisons between individual farms, but do not appear to be correlated with ML selection. No differences in allele frequency were observed at the *Hc-dyf-7* locus in any between-farm comparison. When allele frequencies were pooled for ML− and ML+ groups, there were no statistically significant differences at any locus and any putative ‘selected’ alleles identified in individual farm comparisons were of comparable frequencies in the ML− group and the ML+. Thus, allele frequency analysis found no consistent evidence for selection on any of the candidate genes that is associated with ML treatment.

### 3.5 FST outlier analysis

FST outlier analysis can be used to identify any locus showing a greater genetic distance than expected from the distribution of neutral markers (Beaumont and Nichols, 1996), which would be suggestive of selection. Based on an initial simulation with all loci, putative markers under selection (outside the 95% confidence interval) were discarded and the mean neutral FST distribution was estimated with the remaining neutral markers only (Antao et al., 2008). Individual FST values for all loci were then compared with the neutral FST distribution to identify outliers. Analysis of the entire dataset (either as four distinct farm populations or as two populations grouped by ML treatment history) found no locus under positive selection. All loci behaved as neutral markers, with the exception of *Hc-dyf-7*, which was identified as a candidate locus under balancing selection in the four farm population comparison only. In pairwise comparisons between farm populations, no loci appeared under positive selection, other than *Hc-glc-5* in a comparison of F102− and F5+ only. In all other comparisons all loci behaved as neutral markers, other than *Hc-dyf-7*, which again was identified as a candidate locus under balancing selection in five out of the six between-farm comparisons. Microsatellite 8a20 appeared to be under balancing selection in a comparison of F102− and F101+ only. Again, this analysis shows no consistent evidence for selection on any of the candidate genes that is associated with ML treatment.

### 3.6 Linkage disequilibrium analysis

Since there is reason to believe that ML resistance is multi-genic, LD analysis was undertaken to test for non-random association of alleles at different loci. LD was measured using the Expectation-Maximum (EM) algorithm combined with a likelihood ratio test (Excoffier et al., 2005). In pairwise comparisons of the four candidate gene loci, none showed evidence of LD in the 47 ML+ individuals. Similarly, neither of the ML selected farm populations showed evidence of LD when analysed individually. Only unselected farm F102− showed significant LD, between *Hc-glc-5* and *Hc-avr-14.*

### 3.7 *Hc-dyf-7* sequencing

**Fig. 3:**
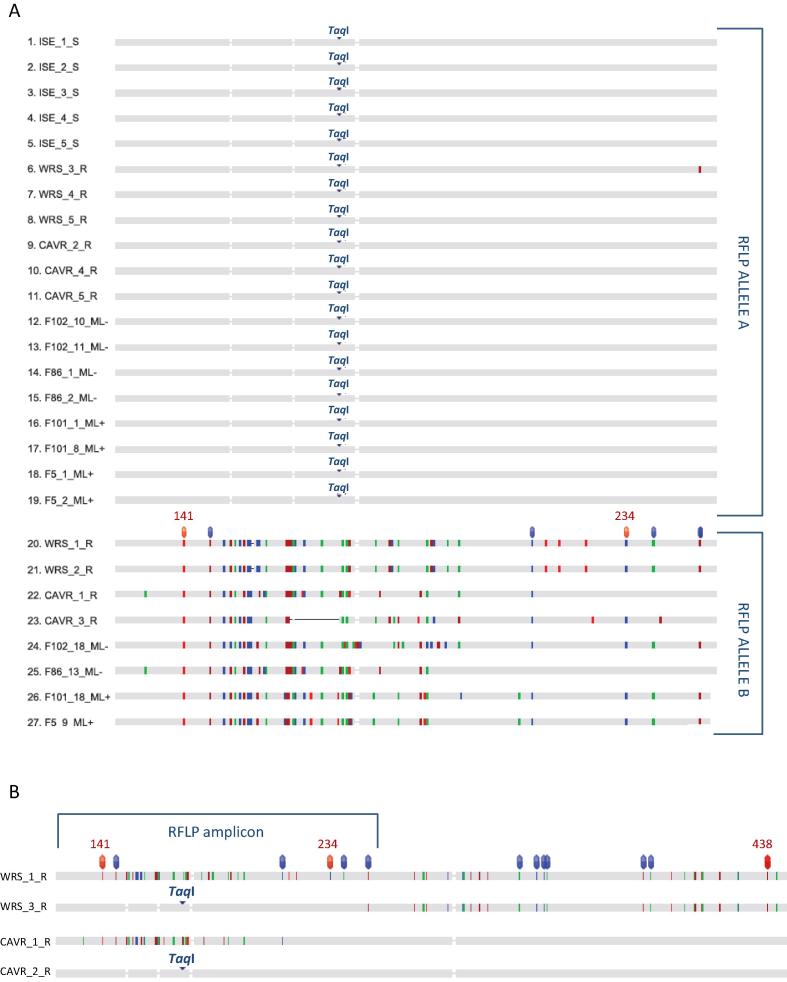
Genomic sequence of *Haemonchus contortus* (*Hc)-dyf-7*. (A) Restriction fragment length polymorphism amplicons. S, macrocyclic lactone-susceptible isolate; R, macrocyclic lactone-resistant isolate, ML−, field population without a history of macrocyclic lactone use; ML+, field population with a history of frequent macrocyclic lactone use. Bullets above alleles highlight single nucleotide polymphisms associated with macrocyclic lactone resistance in Urdaneta-Marquez et al. (2014), with numbered bullets highlighting three single nucleotide polymphisms reported to most reliably predict macrocyclic lactone resistance status (single nucleotide polymphism nucleotide numbers correspond to those in Urdaneta-Marquez et al., 2014). Vertical lines in allele B sequences indicate all single nucleotide polymphisms relative to the allele A reference sequence. Only allele A was present in the five clones from the drug-susceptible laboratory isolate MHco3(ISE), while alleles A and B were present in the five clones from drug-resistant isolates MHco4(WRS) and MHco10(CAVR). Both alleles A and B were present in all farm populations, regardless of anthelmintic use history. WRS_3_R and CAVR_1_R appear to represent recombinant A/B and B/A alleles, respectively. Their un-nested (695 bp) amplicon sequences are shown in B, which extends to the thirteenth single nucleotide polymphism marker associated with macrocyclic lactone resistance in Urdaneta-Marquez et al. (2014). F86_13_ML- may be a B/A recombinant also. The Restriction fragment length polymorphism screen does not differentiate these recombinant alleles.

A striking feature of the *Hc-dyf-7* locus RFLP analysis was that allele A was almost at fixation in the four farm populations, with allele B present at a low frequency in all (Fig. 2). To investigate the lack of diversity at the sequence level, a small subset of *Hc-dyf-7* A and B alleles from all farms were cloned and sequenced: the 343 bp genomic region spanned from exon 2 to exon 3 and included six of the SNP loci reported to predict ML susceptibility or resistance (Urdaneta-Marquez et al., 2014). There was absolute conservation (100% nucleotide identity) in genomic sequences for allele A, whether isolated from ML− or ML+ farms, and none of the polymorphisms associated with ML resistance were present (Fig.3A). In contrast there was some sequence diversity in allele B (83.5–92.2% nucleotide identity) with each allele from the four farms being distinct. All six of the SNPs associated with ML resistance were present in allele B sequences from F102−, F101+ and F5+, which included two of the three major SNPs (positions 141 and 234) defined as the most reliable markers for ML resistance. Only the first two SNPs (one of which was position 141), were present in the allele B sequence from F86−, which appears to be a B/A recombinant allele (see below).

To further explore sequence diversity at the *Hc-dyf-7* locus, the same region was sequenced in a small number of L3s from three laboratory isolates (MHco3(ISE), MHco4(WRS) and MHco10(CAVR)) (Fig.3A). All sequences from the ML-susceptible isolate MHco3(ISE) were the same and represent allele A. In the ML-resistant isolates MHco4(WRS) and MHco10(CAVR), allele A was present with allele B. The genomic sequence of allele A was absolutely conserved in all isolates (100% nucleotide identity), except for a single putative A/B recombinant (WRS_3, containing one of the six SNPs in allele B) and a putative B/A recombinant (CAVR_1, containing three of the SNPs in allele B). To provide further evidence that the WRS_3 and the CAVR_1 sequences were recombinants, a longer region of the gene was sequenced from these two clones, together with representative clones of A and B alleles (Fig.3B). These results confirm that allele B contains all 13 of the SNPs in this region which are reported to correlate with ML resistance, including the three which are considered to be the most reliable markers, and that allele A has none of these. The putative recombinants contained a combination of regions from both alleles.

## 4 Discussion

The aim of this work was to test the importance of candidate ML resistance genes in UK field populations of *H. contortus*. Identifying comparable ML− and ML+ farm populations was challenging, as our samples were collected from commercial enterprises and most UK farmers have used all three of the major classes of anthelmintic in their flocks to try to maintain production (Burgess et al., 2012). However, the four most suitable populations were identified from 118 farms and 23–24 L1s were genotyped from each. While we had no access to phenotypic data for the farm populations, evidence of selection should be detectable at an earlier stage than major changes in population phenotype, and the 6 year treatment histories of the ML+ farms suggested clinically relevant selection pressure had been applied. Both ML+ farms treated ewes with MLs every year (totalling six and 12 treatments) and both used a long acting ML at lambing, which is known to promote resistance by limiting refugia – particularly in the UK where *H. contortus* does not overwinter on pasture. The first reports of IVM-resistant *H. contortus* on farms in South Africa appeared 3 years after the drug was licensed for use in sheep; in one case, after a history of only three treatments and the second after 11 treatments (van Wyk and Malan, 1988). Thus, based on the number of treatments and length of treatment, we expect our ML+ farm populations to have undergone strong selection.

The four candidate genes analysed had previously been associated with ML resistance in various *H. contortus* laboratory strains or field isolates. High levels of genetic diversity, both in terms of allele richness and sequence variation, were identified at the genomic loci of *Hc-lgc-37*, *Hc-glc-5* and *Hc-avr-14*, which is consistent with studies examining other genetic loci in UK field populations of *H. contortus* (Redman et al., 2015). In contrast, very little genetic diversity was observed at the *Hc-dyf-7* locus.

In this study, there was no correlation between ML selection and genotype at *Hc-lgc-37*, *Hc-glc-5* or *Hc-avr-14*. We identified what appeared to be selection for a single allele at the *Hc-lgc-37* and *Hc-glc-5* loci in certain pairwise ML− and ML+ farm comparisons, which is consistent with work by Blackhall et al., 1998a, Blackhall et al., 2003. However, the lack of selection at the same loci in different but equivalent farm comparisons and the high frequencies of the putative selected alleles in other ML− populations, suggests these genes are not consistently selected in the field. Williamson et al. (2011) compared the cDNA sequences of *Hc-lgc-37*, *Hc-glc-5* and *Hc-avr-14* in two *H. contortus* isolates differing in ML resistance phenotype, and similarly found a high degree of genetic polymorphism in all three genes, within and between populations, but no evidence of selection for previously reported coding differences. These findings suggest that either different mechanisms are important on different farms and in different isolates, or that these loci are not under ML selection.

*Hc-glc-5* resides on the same chromosome as the β-tubulin isotype 1 locus, which was subject to a hard sweep in F86− and F101+ (Redman et al., 2015). These loci are likely to be separated by at least 10 Mb, as they are both encoded in the middle of large scaffolds, so recombination would be expected to break up any association. However, if insufficient time had elapsed since the selective sweep, it is possible that *Hc-glc-5* could be included, which would be consistent with the lack of HWE at this locus in these populations only.

Relative to the three other loci, the absolute conservation of genome sequence in *Hc-dyf-7* allele A was striking. Despite the presence of putative ML resistance allele B on farms with 6 years of ML treatment, there was no evidence for selection occurring. Sequencing a small subset of A and B alleles from the four farms found complete consistency with RFLP allele calls, but did detect a single B/A recombinant, called as allele B, on a ML− farm (Fig.3A). If the reverse was true and there were some undetected A/B recombinants, called as allele A, it is possible these could be relevant to ML resistance status.

It is also possible that the small sample size has limited our power to detect more subtle changes in allele frequency. While the primary reports associating the candidate genes with ML resistance typically used similar sample sizes (Blackhall et al., 1998a, Blackhall et al., 2003) and the *Hc-dyf-7* mutation was proposed to require between one and three single worm genotypes to diagnose ML resistance (Urdaneta-Marquez et al., 2014), the size and genetic diversity of field populations may undermine attempts to compare such small numbers of individuals. However, recent analysis of the β-tubulin isotype-1 locus in similar numbers of individuals from the same farm populations identified clear signatures of selection (Redman et al., 2015), demonstrating that it is possible to detect selective sweeps where the resistant alleles are known.

While there was no correlation between ML selection and genotype in all farm comparisons in this study, it is possible that different populations have different mechanisms of ML resistance. This could be either a stochastic effect based on the genetic composition of each population or a product of differing drug treatment regimes. While the treatment histories suggest clinically relevant selection pressure has been applied to the ML+ farms, it is also theoretically possible that a particular management practice that was not specified in the survey could reduce or alter the selection pressure on these farms. However, the lack of evidence for selection at any of the candidate gene loci, despite 6 years of ML treatment, does raise doubts as to the appropriateness of these loci for detecting resistant worms in the field.

A number of factors can confound candidate gene analysis and may underlie the plethora of genes associated with ML resistance to date. This study has highlighted a number of considerations for researchers using similar approaches. Nematode populations have high levels of genetic variation, even within populations, and the study of a single locus in isolation is likely to detect differences which may be wrongly interpreted as a positive correlation with the trait of interest. More powerful approaches, using genome-wide or whole genome sequencing, are likely to be required. Working with field isolates is subject to numerous variables, many of which are difficult to avoid. However, these are the relevant populations for ML resistance research, so it is important to better understand these complexities and develop strategies to address them.

## Figures and Tables

**Table 1:** Anthelmintic use in ewes. F5+ and F101+ frequently use macrocyclic lactones, F86− and F102− rarely/never use macrocyclic lactones. Farm management data from Burgess et al. (2012).

Farm	Drenches per year	Anthelmintic use in ewes						Dose to	Quarantine drench	Long acting drug at lambing	Confirmed AR	Dose and move	ML to treat ectoparasites
		2008	2007	2006	2005	2004	2003						
F5+	1	ML	ML	ML	ML	ML	ML	Estimate	ML	ML	No	No	No
F101+	2	BZ, LV, ML	LV, ML	LV, ML	LV, ML	LV, ML	LV, ML	Estimate	ML	ML	No	Yes	No
F86-	2	LV		LV	BZ	LV	BZ	Heaviest	BZ, LV, ML	No	BZ	No	No
F102-	2	BZ	BZ	BZ	BZ	LV	LV	Heaviest	No	No	No	Yes	No

BZ, Benzimidazole; LV, Levamisole; ML, macrocyclic lactone.

**Table 2:** Fixation index values for pairwise farm comparisons. F5+ and F101+ frequently use macrocyclic lactones, F86− and F102− rarely/never use macrocyclic lactones. Fixation index with 10 microsatellites shown in column 2 is from Redman et al. (2015).

Farm comparison	FST with 10 microsatellites (with null correction) *n* = 30	FST with 4 microsatellites (with null correction) *n* = 23/24	FST with 4 microsatellites and 4 candidate gene loci *n* = 23/24
F86− and F101+	0.0115 (0.0121)	0.0122 (0.0208)	0.0143
F86− and F5+	–	0.0158 (0.0180)	0.0088
F102− and F101+	0.0453 (0.0409)	0.0231 (0.0193)	0.0365
F102− and F5+	–	0.0165 (0.0053)	0.0362

F86− and F102−	0.0514 (0.0459)	0.0363 (0.0237)	0.0670
F101+ and F5+	–	0.0165 (0.0135)	0.0221

**Table 3:** *X*^2^ analysis of candidate gene allele frequencies. F5+ and F101+ frequently use macrocyclic lactones, F86− and F102− rarely/never use macrocyclic lactones. Comparisons are shown for individual farms and for farms pooled by macrocyclic lactone use (ML− and ML+).

Locus	Comparison	X^2^	*P*
*Hc-lgc-37*	F102− and F101+	12.29	0.006^a^
	F102− and F5+	1.91	0.591
	F86− and F101+	2.89	0.409
	F86− and F5+	6.74	0.081
	ML− and ML+	2.17	0.540
	F102− and F86−	14.67	0.002^a^
	F101+ and F5+	5.09	0.165

*Hc-glc-5*	F102− and F101+	4.05	0.130
	F102− and F5+	14.85	0.001^a^
	F86− and F101+	6.27	0.043^a^
	F86− and F5+	0.41	0.816
	ML− and ML+	1.52	0.470
	F102− and F86−	16.28	0.000^a^
	F101+ and F5+	7.01	0.030^a^

*Hc-avr-14*	F102− and F101+	14.35	0.006^a^
	F102− and F5+	7.11	0.130
	F86− and F101+	2.83	0.587
	F86− and F5+	2.80	0.592
	ML− and ML+	3.84	0.428
	F102− and F86−	10.42	0.034^a^
	F101+ and F5+	8.75	0.068

*Hc-dyf-7*	F102− and F101+	0.62	0.430
	F102− and F5+	0.00	0.964
	F86− and F101+	0.11	0.740
	F86− and F5+	0.26	0.609
	ML− and ML+	0.06	0.814
	F102− and F86−	0.21	0.645
	F101+ and F5+	0.71	0.398

a *P* < 0.05 was considered statistically significant.

## Appendix A Supplementary data

Supplementary Fig. S1 Restriction fragment length polymorphism (RFLP) allele scoring approach for *Haemonchus contortus (Hc)-glc-5*. (A) Genomic sequences of the *Hc-glc-5* locus in 20 clones from a pool of 20 F102− L1. Coloured vertical bars represent single nucleotide polymphisms (SNPs) relative to consensus and the *Taq*I cut sites are highlighted. (B) RFLP digests at the *Hc-glc-5* locus for 10 individual L1s, with their corresponding genotypes recorded above.

Supplementary Fig. S2 *Haemonchus contortus (Hc)-avr-14* clones from F102−. Twenty clones were sequenced, in both directions, from a pool of 20 L1s to assess baseline levels of polymorphism and design the allele-specific PCR assay. Coloured vertical bars represent single nucleotide polymphisms relative to consensus. Blue arrows represent primer binding regions; primer F4 is universal, primer RsA will only amplify allele A, and primer RsnA will only amplify non-A alleles.

Supplementary Fig. S3 *Haemonchus contortus (Hc)-lgc-37, Hc-glc-5* and *Hc-avr-14* clones from F102−. Twenty clones were sequenced, in both directions, from a pool of 20 L1 to assess baseline levels of polymorphism and design restriction fragment length polymorphism or allele-specific PCR assays. Coloured vertical bars represent single nucleotide polymphisms relative to consensus. High levels of genetic diversity, both in terms of allele richness and sequence variation, are apparent.

Supplementary Table S1 Primer sequences and genomic regions for candidate macrocyclic lactone (ML) resistance genes in *Haemonchus contortus* (*Hc*).
