# Supplementary material for: Analysis of putative resistance gene loci in UK field populations of Haemonchus contortus after 6 years of macrocyclic lactone use
Source: Int J Parasitol. 2016 Sep;46(10):621–30. doi: 10.1016/j.ijpara.2016.03.010 (PMC5011429; doi:10.1016/j.ijpara.2016.03.010)
Supplement: Supplementary Fig. S1 — Restriction fragment length polymorphism (RFLP) allele scoring approach for Haemonchus contortus (Hc)-glc-5. (A) Genomic sequences of the Hc-glc-5 locus in 20 clones from a pool of 20 F102− L1. Coloured vertical bars represent single nucleotide polymphisms (SNPs) relative to consensus and the TaqI cut sites are highlighted. (B) RFLP digests at the Hc-glc-5 locus for 10 individual L1s, with their corresponding genotypes recorded above. [file mmc1.pptx]

## Slide 1
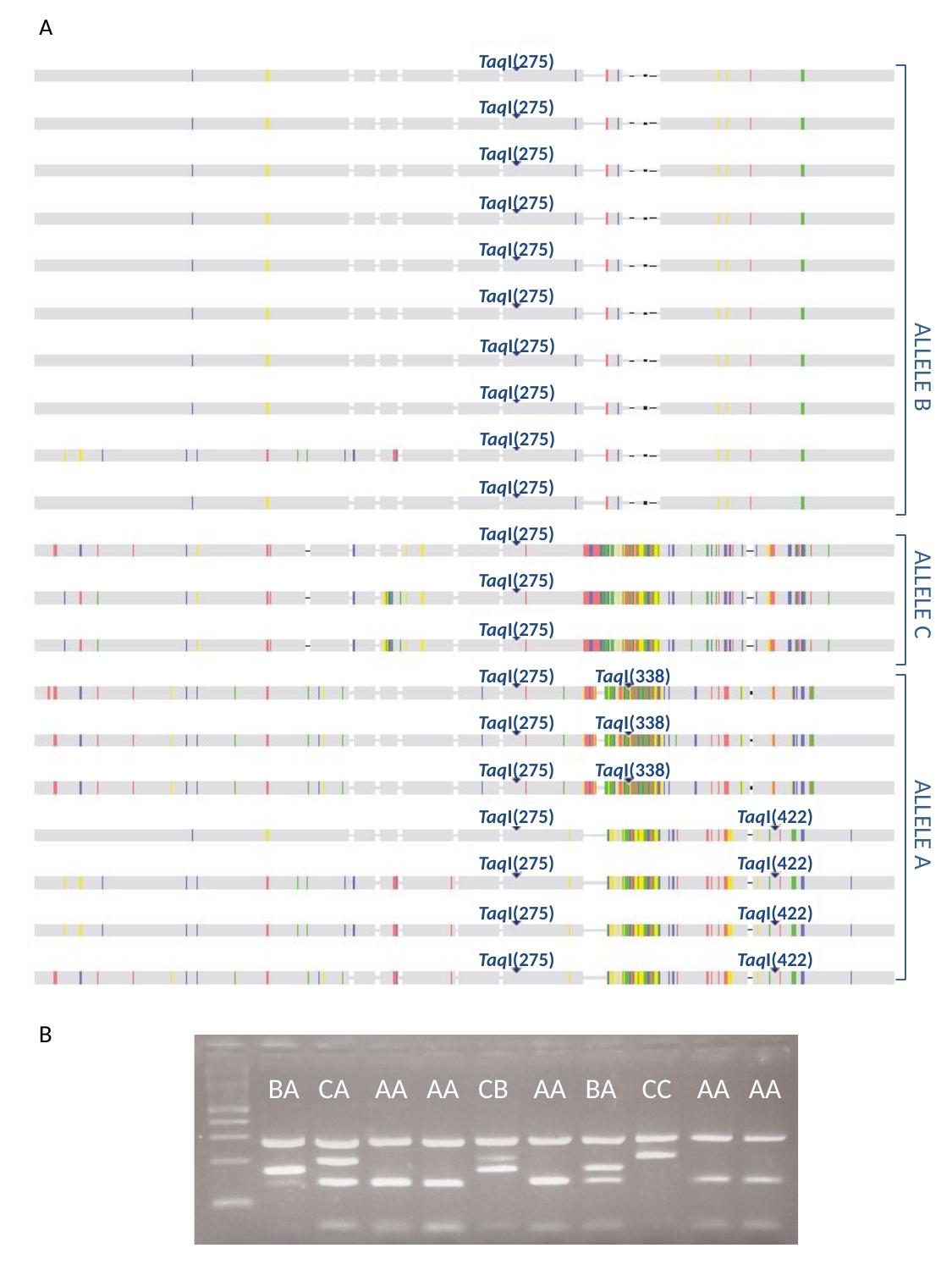

A
B
TaqI(275)
TaqI(275)
TaqI(275)
TaqI(275)
TaqI(275)
TaqI(275)
TaqI(275)
TaqI(275)
TaqI(275)
ALLELE B
TaqI(275)
TaqI(275)
TaqI(275)
ALLELE C
TaqI(275)
TaqI(275) TaqI(338)
TaqI(275) TaqI(338)
TaqI(275) TaqI(338)
TaqI(275) TaqI(422)
TaqI(275) TaqI(422)
ALLELE A
TaqI(275) TaqI(422)
TaqI(275) TaqI(422)
 BA CA AA AA CB AA BA CC AA AA
