# Supplementary figures and images for: Analysis of putative resistance gene loci in UK field populations of Haemonchus contortus after 6 years of macrocyclic lactone use
Source: Int J Parasitol. 2016 Sep;46(10):621–30. doi: 10.1016/j.ijpara.2016.03.010 (PMC5011429; doi:10.1016/j.ijpara.2016.03.010)

## Slide 1
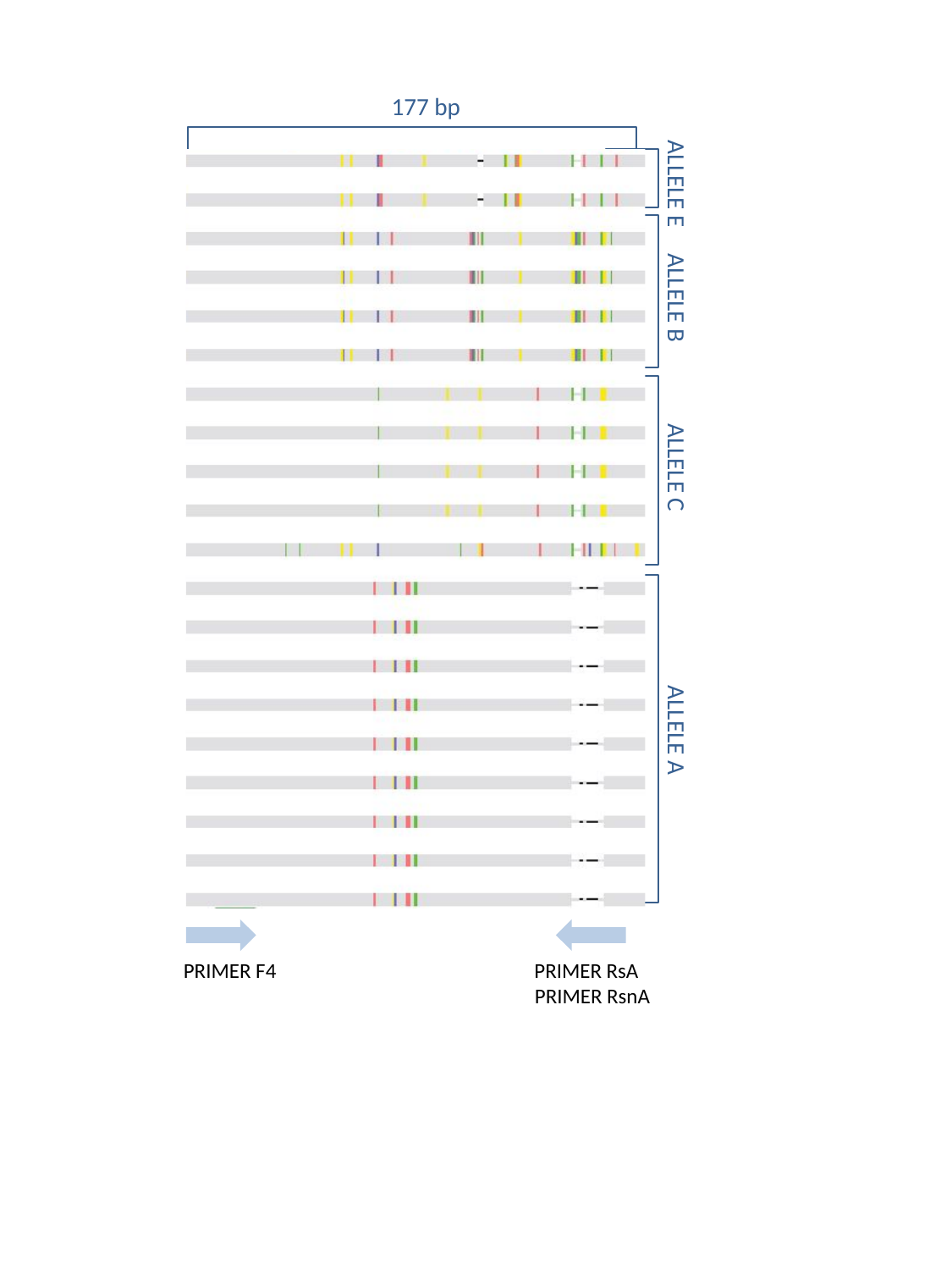

177 bp
ALLELE E
ALLELE B
ALLELE C
ALLELE A
 PRIMER F4 	 PRIMER RsA
		 PRIMER RsnA

Supplement: Supplementary Fig. S2 — Haemonchus contortus (Hc)-avr-14 clones from F102−. Twenty clones were sequenced, in both directions, from a pool of 20 L1s to assess baseline levels of polymorphism and design the allele-specific PCR assay. Coloured vertical bars represent single nucleotide polymphisms relative to consensus. Blue arrows represent primer binding regions; primer F4 is universal, primer RsA will only amplify allele A, and primer RsnA will only amplify non-A alleles. [file mmc2.pptx]

## Slide 1
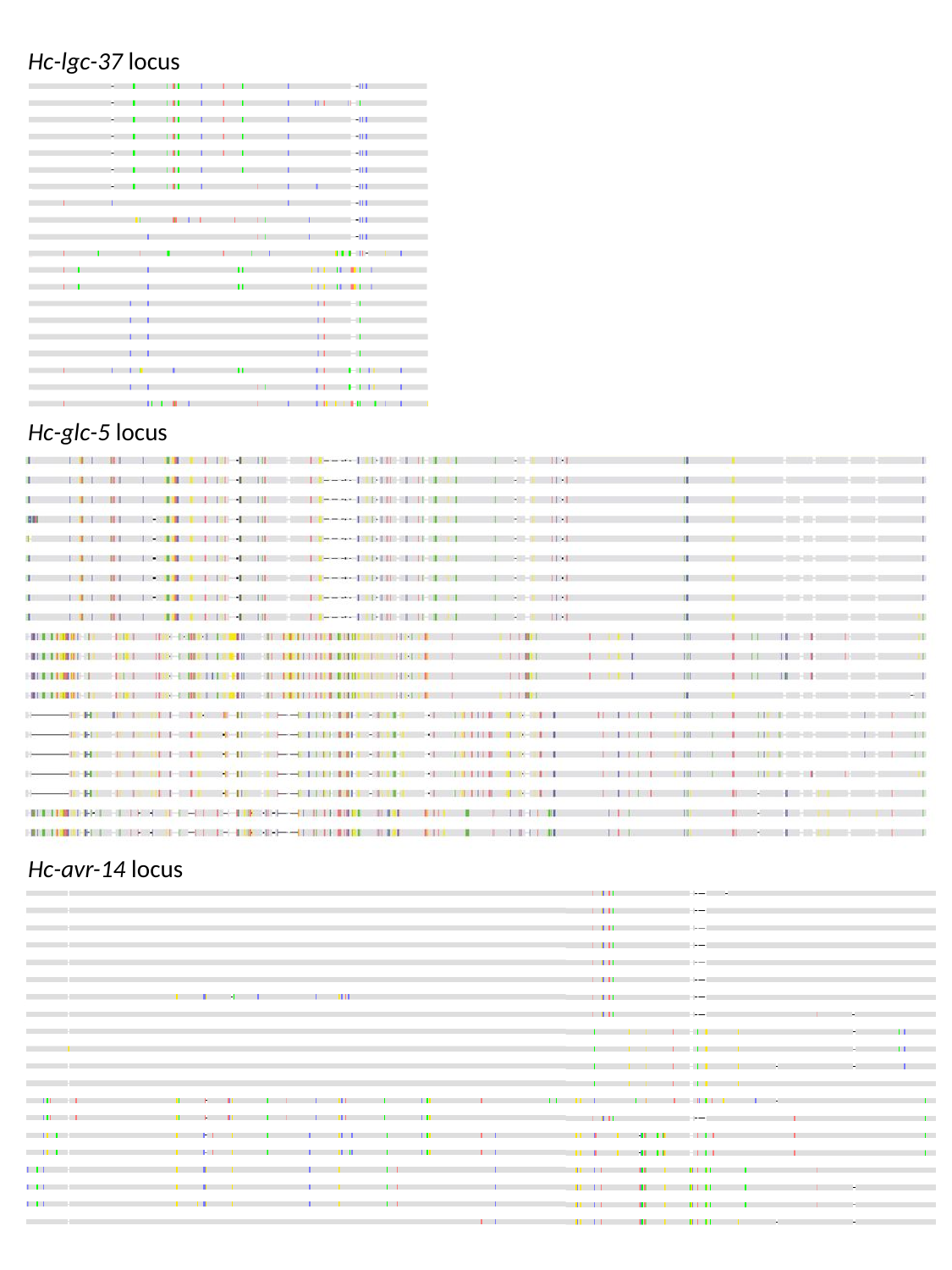

Hc-lgc-37 locus
Hc-glc-5 locus
Hc-avr-14 locus

Supplement: Supplementary Fig. S3 — Haemonchus contortus (Hc)-lgc-37, Hc-glc-5 and Hc-avr-14 clones from F102−. Twenty clones were sequenced, in both directions, from a pool of 20 L1 to assess baseline levels of polymorphism and design restriction fragment length polymorphism or allele-specific PCR assays. Coloured vertical bars represent single nucleotide polymphisms relative to consensus. High levels of genetic diversity, both in terms of allele richness and sequence variation, are apparent. [file mmc3.pptx]
