# Supplementary material for: Analysis of putative resistance gene loci in UK field populations of Haemonchus contortus after 6 years of macrocyclic lactone use
Source: Int J Parasitol. 2016 Sep;46(10):621–30. doi: 10.1016/j.ijpara.2016.03.010 (PMC5011429; doi:10.1016/j.ijpara.2016.03.010)
Supplement: Supplementary Table S1 — Primer sequences and genomic regions for candidate macrocyclic lactone (ML) resistance genes in Haemonchus contortus (Hc). [file mmc4.docx]

|  | Tm (^o^C) | Product size (bp) | Genomic region |
| --- | --- | --- | --- |
| *Hc-lgc-37* (*hg1* (Blackhall et al., 2003)) |  |  |  |
| F: GGTGATGTCATGGGTGTC | 56 |  |  |
| R: TTGCTGCGAATACGAATC | 51.4 | 305 | exon 8 to exon 10 |
|  |  |  |  |
| *Hc-glc-5* |  |  |  |
| newF: GCAGTATCGTGGGTGTCTT | 56.7 |  |  |
| R1: CTTGTCGTGAGTCTGGATTC | 57.3 | 1080 | exon 7 to exon 10 |
| F1: CCGGCTCGGGTTACGCT | 60 | 465 | exon 8 to exon 10 |
|  |  |  |  |
| *Hc-avr-14* |  |  |  |
| F2: CAATTCCGTCCCTCTGGCG | 61 |  |  |
| Rnest: CGAACCTAACGCTGTAAAGG | 57.3 | 784 | exon 1 to exon 4 |
| F4: GCCGACCAGTCACAACAG | 58.2 |  |  |
| RsA: GCCAACCGAGAGTAGCCA | 58.2 | 177 | exon 3 to intron 3 |
| RsnA: GCCAACCGAAAGCAGGAG | 58.2 | 177 | exon 3 to intron 3 |
|  |  |  |  |
| *Hc-dyf-7* |  |  |  |
| F4: GTGTCATGCTCGTCGGG | 57.6 |  |  |
| R4: CTCTACTTTCGGTTTCGGCT | 57.3 | 695 | exon 2 to exon 5 |
| R6: CAACAGATGCTAACAAACCAG | 55.9 | 343 | exon 2 to exon 3 |

**Supplementary Table S1.** Primer sequences and genomic regions for candidate macrocyclic lactone (ML) resistance genes in *Haemonchus contortus* (*Hc*).

**References**

Blackhall, W.J., Prichard, R.K., Beech, R.N., 2003. Selection at a gamma-aminobutyric acid receptor gene in *Haemonchus contortus* resistant to avermectins/milbemycins. Mol Biochem Parasitol. 131, 137-145.
